# Effect of educational intervention based on health belief model on mothers monitoring growth of 6–12 months child with growth disorders

**DOI:** 10.1186/s12887-022-03593-8

**Published:** 2022-09-23

**Authors:** Ali Khani Jeihooni, Fatemeh Mohammadkhah, Fatemeh Razmjouie, Pooyan Afzali Harsini, Fariba Sedghi Jahromi

**Affiliations:** 1 Nutrition Research Center, Department of Public Health, School of Health, Shiraz University of Medical Sciences, Shiraz, Iran; 2 Department of Community Health, child nursing and aging, Ramsar School of Nursing, Babol University of Medical Sciences, Babol, Iran; 3 Department of Health Promotion and Aging, School of Health, Shiraz University of Medical Sciences, Shiraz, Iran; 4 Department of Public Health, School of Health, Kermanshah University of Medical Sciences, Kermanshah, Iran

**Keywords:** Children, Mothers, Health Belief Model (HBM), Growth Disorders

## Abstract

**Background:**

Maternal education is one of the main ways to improve children's nutritional behaviors and development. The purpose of this study is to investigate the effect of educational intervention based on Health Belief Model (HBM) on mothers monitoring growth of 6–12 months child with growth disorders in Ghirokarzin city, Fars Provonce, Iran.

**Methods:**

This quasi-experimental study was conducted on mothers of 6–12 months children with growth disorders of Ghirokarzin city, Fars province, Iran in 2021–2022. One hundred twenty mothers of 6–12 months child with growth disorders in Ghirokarzin city were selected using random sampling method and were divided into two groups of intervention (60) and control (60). The experimental group received training on the HBM constructs. Both groups completed the questionnaire before and three months after.intervention. A questionnaire beased on Health Belief Model constructs were used to collect information. The data was analyzed with SPSS 22 software using paired t-tests, Chi-square tests, and independent t-tests, with a significance level of 0.05.

**Results:**

Three months after the educational intervention, the experimental group showed a significant increase in terms of knowledge, HBM constructs, weight of the children and feeding behavior.

**Conclusion:**

This study showed the educational intervention based on the HBM improved the knowledge and feeding behavior of mothers and improved Growth Disorders of child. Hence, this model can act as a framework for designing and implementing educational interventions for prevention of growth disorders in children.

## Background

Growth disorder is a term that refers to stopping or slowing down the growth of children, being manifesting in three forms of thinness, short stature, and low weight [1].Pediatric growth disorders originate from a wide variety of etiologies and no definite pathogenesis is ascertained [2] and comprise the largest group of referrals to a pediatric endocrinology consultation service [3]. Nutritional deficiencies lead to several complications such as patients’ clinical outcomes, quality of life, body function, and autonomy. According to World Health Organization, Around 45% of deaths among children under 5 years of age are linked to undernutrition. These mostly occur in low- and middle-income countries. At the same time, in these same countries, rates of childhood overweight and obesity are rising.

Malnutrition is a multifaceted problem which is caused by a shortage of nutritionists, poverty, lack of access to adequate food, nutritional ignorance, and poor eating habits. However, the experiences in other countries indicate that successful reduction of malnutrition hinges upon improving eating knowledge and creating proper eating behaviours [4].

Growth disorder is usually so slow that it is not detectable to the mother by observation, and sometimes health workers have difficulty in the diagnosis And cognitive delay was associated with an increased prevalence of exposure to undernutrition In both middle-income and low-income countries, significant that were more than twice as likely than their peers to be exposed to severe underweight, severe wasting and severe stunting [5].

The most common age for growth disorder is 6-12 months since the child starts to feed on other food groups than mere mother’s milk [6].

Usually, children weigh well up to 6 months and their growth is in accordance with the reference curves, however, the prevalence of underweight increases with age. The most common age for growth disorder is 6-12 months [7].

The first year of a child's life is a very important and irreversible period in growth. Malnutrition leads to irreversible effects on a child's physical growth, brain development and health [8].

In general, malnutrition has both short-term and long-term consequences. Thus, child malnutrition errors are fundamental concerns for survival, health development, and economic and social capabilities [7]. Malnutrition disrupts body's defense mechanism against infections and dealing with them. As a result, it increases the prevalence, severity and duration of common childhood diseases (such as diarrhoea, respiratory infections and measles), decelerates recovery and increases mortality. About 45% of under-5 mortality in middle-income and low-income countries is mainly related to malnutrition [9]. In malnourished children and suckling, the likelihood of repeated hospitalization at great expense on the one hand and the possibility of growth delay and irreparable damage to cognitive abilities on the other hand are greatly increased [10]. Studies have shown the adverse effects that even mild to moderate forms of malnutrition can have an effect on health, that is, without low weight and short stature, by reducing children’s mobility, motivation, curiosity, and their involvement with environment, they disrupt the cognitive development [9]. The child growth disorder is so important that in 2015, all member states of the World Health Organization committed themselves to end all forms of malnutrition by 2030 and to achieve the internationally agreed goals for the short stature and low weight of children under 5 years of age by 2025 [11]. Recent evidences suggest that developing countries are entering a phase of an eating disorder epidemic [11].

The growth disorder has different causes, ranging from Social, economic factors and access to health facilities [12].

It has been proven that the cause of 46% of growth disorders is non-organ and is related to the feeding behavior of the mother [13]. Maternal education is one of the main ways to improve children's feeding behaviors and growth. Findings of various studies have shown the effective role of education in mother’s awareness of child weight improvement [14].

In order for education to have the desired effect on feeding behaviors, it is necessary to provide education based on appropriate theories, because theories have the potential to increase the effectiveness of health education programs [15].

In health education, there are models designed to be used as a practical aid effective educational program, being able to change the unhealthy behaviors [16]. One of these models is the health belief model [17].

According to the health belief model, the adoption of healthy behavior depends on one’s belief in a particular health problem, accepting its reality, being sensitive to its impact on one’s health feeling its threat (perceived sensitivity), recognizing the problem as a critical health issue, understanding its various effects on different dimensions of one’s physical, social, mental and economic health (perceived severity), being convinced by the cues they receive from the environment (cues to action) that preventive activities, while very useful and practical, are economically viable (perceived benefits), and finding barriers against the behavior less costly than its benefits (perceived barriers) [18].

In various studies, the model has been used for children with growth disorders, including Navabi et al. [19] and Hazavei et al. [6].

Considering the importance of growth disorder in children, the prominent role of mother's education as one of the main ways to improve nutritional behaviors and growth disorders and the existence of theories capable of increasing the effectiveness of health education programs such as the Health Belief Model the present study aimed to investigate the effect of educational intervention based on the health belief model on mothers monitoring the growth of 6–12 months child with growth disorders in rural health centers of Ghirokarzin city, Fars Provonce, Iran.

## Methods

This quasi-experimental study was conducted on mothers of 6–12 months children with growth disorders under the auspices of rural health centers of Ghirokarzin city, Fars Province, Iran during 2021–2022. According to a study by Aghdasi et al. [20] and considering 95% confidence and 80% test power, the sample size was determined 100 (50 in the experimental group and 50 in the control group) which, taking data loss into account, was increased up to 120.

$$n=\frac{\left(Z_{1-{\displaystyle\frac\alpha2}}+Z_{1-\beta}\right)^2\left(\delta_1^2+\delta_2^2\right)}{\left(\mu_1-\mu_2\right)^2}$$

First, from among all rural health centers in Ghirokarzin(6 centers), 2 centers were selected using randomly. Then, participants were recruited using a convenience sampling method from each center(60 mothers in each group)**.**

### Inclusion and exclusion criteria

Inclusion criteria were mothers of at least one 6–12 months child with growth disorders **(**weight disorder**)**, Ability to read and fill out questionnaires, willingness to participate in the study, not being educated or employed in medical sciences, being directly responsible for feeding the child, presence of both parents in the family, having a fixed income, and no genetic, Start of complementary feeding at 6 months, incurable disease, major organ defect or problems such as hyperactivity in children.

Exclusion criteria were unwillingness to participate in the study, absence from more than one training session, relocation of the mother and lack of access to her, acute illness of child during the research period.

### Data collection tool

The tool used in this study was a questionnaire made by researchers in accordance with other similar studies [6, 14, 19–22].

The data collection tool consisted of demographic characteristics (child gender, child age, maternal age, parents’ occupation, parents’ education, number of children, and monthly household income), health belief model (HBM) questionnaire and mothers’ feeding behaviors questionnaire. HBM questionnaire includes 10 multiple-choice questions on knowledge (the correct answer had a score of 1 and the wrong or “no idea” had a zero score. The range of scores was from 0 to 10 points), 6 questions on perceived sensitivity in a 5-point Likert scale (the range of scores was from 6 to 30 points), 6 questions on perceived severity in a 5-point Likert scale (the range of scores was from 6 to 30 points), 8 questions on perceived benefits in a 5-point Likert scale (the range of scores was from 8 to 40 points), 7 questions on perceived barriers in a 5-point Likert scale (the range of scores was from 7 to 35 points), 8 question on self-efficacy in a 5-point Likert scale (the range of scores was from 8 to 40 points), and 6 questions on cues to action in a 5-point Likert scale (the range of scores was from 6 to 30 points). The mothers’ feeding behaviors questionnaire also included 12 items (the range of scores was from 0 to 12 points).

In order to evaluate the validity of questionnaire, the impact index item higher than 0.15 were considered, and based on the exploratory factor analysis; they were classified into eight factors. In order to determine the face validity, a list of items was checked by 40 women with demographic, economic, social, and other characteristics similar to the target population. In order to determine the content validity, 12 specialists and professionals in health education and promotion (outside the research team) (*n* = 10), nutrition (*n* = 1), and pediatric (*n* = 1) were consulted. Then, based on Lawshe’s table, items with CVR value higher than 0.56 for 12 people were considered acceptable and were retained for subsequent analysis. The calculated values were higher than 0.70 for the items.

The overall reliability of tool based on Cronbach’s alpha is 0.89. Cronbach’s alpha is 0.88 for knowledge, 0.87 for perceived sensivity; 0.89 for perceived severity, 0.86 for perceived benefits, 0.84 for perceived barriers and 0.88 for self-efficacy. Since alpha values calculated for each of the studied structures are higher than 0.7, it can be said that, the reliability level of tool is acceptable [23–25].

#### Weight measurement scale

The weight of all children studied was measured, recorded and compared before and 3 month after the intervention. In order to measure children's weight, a single calibrated digital scale with a measurement accuracy of 0.1 kg (100 g) was used.

#### Criteria for diagnosing growth disorders

**Fig. 1:**
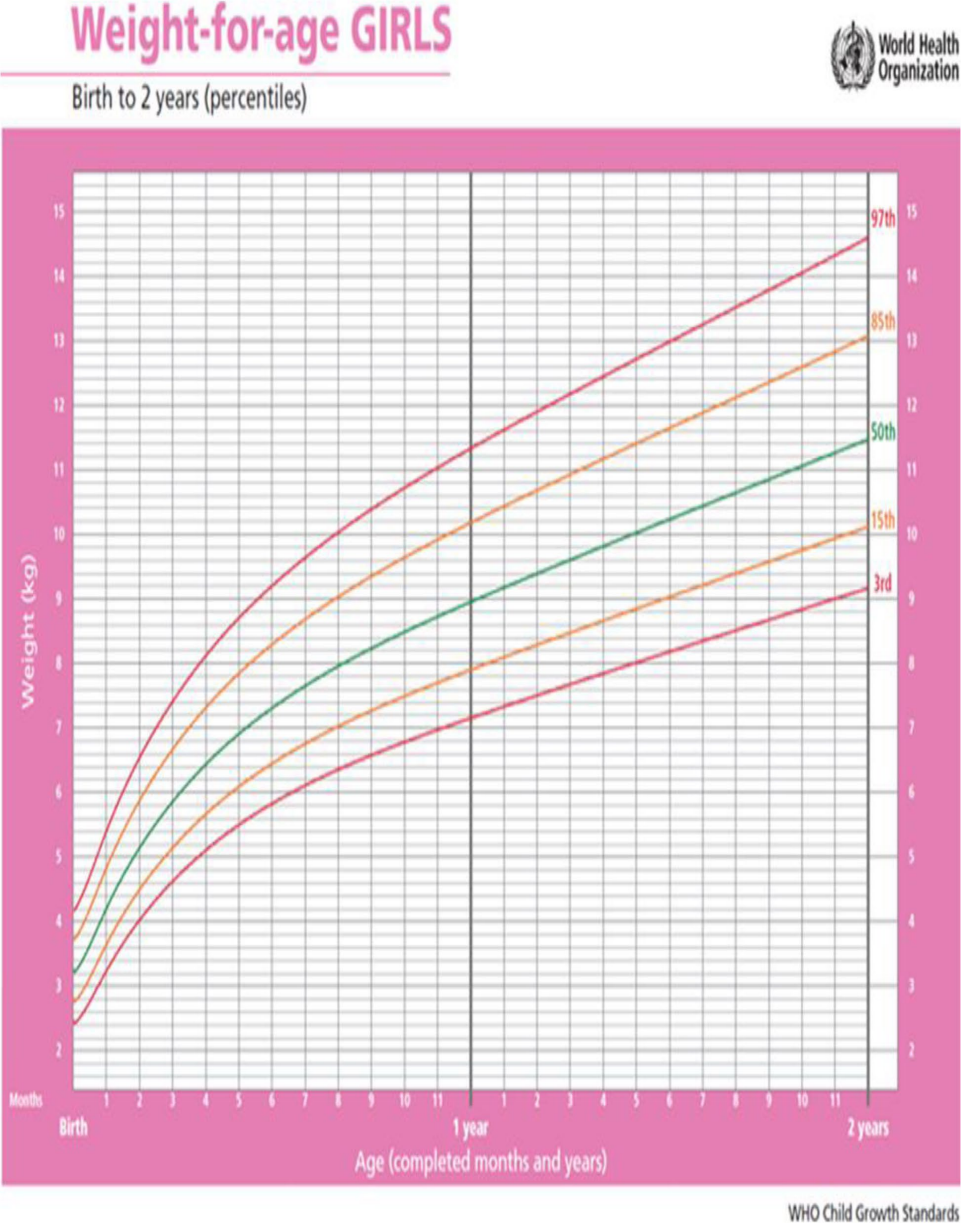
Child weight curve table

After accurate measurement of the child's weight, it was recorded in the electronic file of Child weight curve table. Children eligible for Inclusion criteria and growth disorders [childerens with severe thin (below -3z score), thin (-3z score to -2z score) and children with normal weight range (-2z score to + 1z score) but with unknown growth trend, the process of stopped growth and weight loss of the child)] were included in the study [26] (Fig. 1-sample of Child weight curve). Also mothers were taught about the importance, application, and interpretation of the child's growth curve.

### Educational intervention

After obtaining a written consent from mothers of the 6–12 year old children participating in the present study, the purpose of the study and how to do the work were explained to them The questionnaire was completed by two experimental and control groups(pre-test). Based on the findings of the pre-test, the educational content was developed. In the experimental group, eight 50–55-min training sessions included presentations, questions and answers, group discussions, informative posters, brochures, and video clips(Table 1).

**Table 1:** Description of training sessions held in the experimental group

**Number of sessions**	**The educational content**
1	Familiarity with growth disorders, symptoms, complications and disease diagnosis, risk factors (in order to increase the structure of Knowledge and perceived sensitivity)
2	A doctor of health education and health promotion, a dietitian, and a health care professional led the instruction. Many different topics were discussed during the sessions, including early and late complementary feeding, the causes of infant growth disorder, the frequency of complementary feeding and breastfeeding according to the age of the child, prohibited foods under one year, optimal feeding behavior, child development and its relationship with nutrition, mothers' behaviors when feeding their children, different types of food according to the age of the child, the importance of taking supplements, and the importance of taking supplements. (in order to increase the structure of perceived sensitivity)
3	The mother of an 8-month-old child who had a growth disorder was invited and she spoke to people about the growth disorder and the problems caused by it. In this meeting, it was emphasized to change the positive attitude of people to prevent growth disorders and the role of mothers in proper feeding of children And mothers are well aware of the severe consequences of growth disorders in their children, both in the long- (thinness, short stature, low weight) and in short-term (severe anemia, recurrent infectious diseases, etc.) and they know that growth disorders, particularly chronic disorders, may have grave effects on the life of their child. (in order to increase the structure of perceived severity)
4	The role of nutrition in the prevention of growth disorders, providing a suitable food pattern, changing attitudes and making decisions (in order to increase the structure of perceived benefits).
5	Each woman was also asked to discuss her prior nutrition experiences Also, the mothers discussed with each other about the Perceived barriers of giving proper food to the child and the strategies to deal with it (to reduce the Perceived barriers).
6	During a training session, people were taught about how to prepare several types of nutritious and age-appropriate food for children (porridge-almond butter and soup) with the help of each other, they prepared almond butter (in order to increase self-efficacy structure).
7	All mothers educated by health center staff, physicians, the Internet, and the mass media. (Cues to action)
8	reviewed the contents of the previous meetings and People divided into groups of 8–10 people and discussed and exchanged information and experiences with each other.

All mothers and their wives received informative pamphlets at the end of the courses. Every week, a WhatsApp group was formed to exchange information, and instructive and motivating messages were delivered to the participants. Two virtual follow-up sessions were held for mothers one month and two months after the educational intervention. The questionnaire was completed by both the experimental and control groups three months after the educational intervention. The weight of the children was measured before and three months after the educational intervention. The control group received no training and the necessary care instruction was provided on a regular basis by health care providers and health workers. The control group received an informative booklet at the end of the study.

### Data analysis

Data were analysed by using SPSS22 software through Chi-square, independent t-test, and paired t-test. The effect of the intervention was measured by Mean difference score of knowledge, perceived sensitivity, perceived severity, perceived benefits, perceived barriers, self-efficacy, cues to action and mothers’ feeding behaviors in experimental and control groups before and three months after the educational intervention.

## Results

This study was performed on 120 mothers of 6–12 months children with growth disorders. The mean age of mothers in the experimental and control groups was 30.32 ± 5.26 and 29.75 ± 5.30 years, respectively (*p* = 0.276). The mean age of children in the experimental and control groups was 8.43 ± 1.64 and 8.68 ± 1.44 months, respectively (*p* = 0.284). The mean weight of children in the experimental and control groups was 7338 ± 652.37 and 7582 ± 639.189 g, respectively (*p* = 0.069). The independent t-test did not show a significant difference between the two groups in the mentioned variables(Mother’s occupation, Father’s occupation, Monthly household income, Mother’s education, Father’s education, Children’s genderm and Number of children). Other demographic characteristics did not show significant differences between the two groups (Table 2).

**Table 2:** Frequency distribution of demographic characteristics of experimental and control groups (*n* = 120)

**Variables**		**Experimental group**		**Control group**		***P*-value**
		**Number**	**Percentage**	**Number**	**Percentge**	
Mother’s occupation	Housewife	54	90	52	86.67	0.287
	Employed	6	10	8	13.33	
Father’s occupation	Unemployed	4	6.67	2	3.34	0.194
	Employed	7	11.66	8	13.33	
	Worker	15	25	20	33.33	
	Self-employed	34	56.67	30	50	
Monthly household income	< 30 million Rials	35	58.33	30	50	0.168
	30–50 million Rials	18	30	21	35	
	>50 million Rials	7	11.67	9	15	
Mother’s education	Primary school	8	13.33	5	8.33	0.228
	Secondary school	16	26.67	18	30	
	High school	24	40	28	46.67	
	College	12	20	9	15	
Father’s education	Primary school	9	15	6	10	0.116
	Secondary school	12	20	18	30	
	High school	30	50	21	35	
	College	9	15	15	25	
Children’s gender	Male	36	60	31	51.67	0.152
	Female	24	40	29	48.33	
Number of children	1–2	29	48.33	31	51.67	0.175
	3–4	21	35	24	40	
	> 4	10	16.67	5	8.33	

The results showed no significant difference between the experimental and control groups in terms of knowledge, perceived sensitivity, perceived severity, perceived benefits, perceived barriers, self-efficacy, cues to action and feeding behavior before the educational intervention. Three months after the educational intervention, the experimental group showed a significant increase in each of the mentioned variables except the perceived barriers (Table 3).

**Table 3:** Mean score of knowledge, perceived sensitivity, perceived severity, perceived benefits, perceived barriers, self-efficacy, cues to action and mothers’ feeding behaviors in experimental and control groups before and three months after the educational intervention

**Variable**	**Group**	**Before the intervention**	**3 months after the intervention**	**Mean difference**	***p*-value **
Knowledge	experimental	4.25±0.56	8.18±0.92	+3.93	0.001
	control	4.16±0.74	4.58±0.80	+0.42	0.226
	*p*-value	0.238	0.001		
Perceived sensitivity	experimental	10.34±1.50	26.66±1.48	+16.31	0.001
	control	11.16±1.38	11.94±1.41	+0.78	0.204
	*p*-value	0.218	0.001		
Perceived severity	experimental	10.36±1.28	26.74±1.14	+16.37	0.001
	control	9.28±1.24	10.26±1.22	+0.97	0.188
	*p*-value	0.190	0.001		
Perceived benefits	experimental	14.58±2.43	34.22±2.75	+19.64	0.001
	control	15.12±2.39	16.26±2.42	+1.14	0.181
	*p*-value	0.220	0.001		
Perceived barriers	experimental	28.82±1.64	14.15±1.47	-14.67	0.001
	control	28.23±1.70	27.39±1.68	-0.84	0.176
	*p*-value	0.244	0.001		
Perceived self-efficacy	experimental	18.29±2.46	34.12±2.82	+15.83	0.001
	control	20.08±2.39	22.86±2.40	+2.78	0.152
	*p*-value	0.165	0.001		
Cues to action	experimental	12.62±1.57	25.28±1.46	+12.65	0.001
	control	14.58±1.62	14.88±1.57	+0.29	0.214
	*p*-value	0.144	0.001		
Mothers’ feeding behaviors	experimental	4.32±0.72	10.18±0.83	+5.86	0.001
	control	4.69±0.60	4.78±0.65	+0.09	0.223
	*p*-value	0.202	0.001		

The results of the study showed a significant difference in the mean weight of the children in the experimental group three months after the intervention (*p* < 0.001) (Table 4).

**Table 4:** Mean and standard deviation of child weight in the experimental and control groups before and three months after the educational intervention

**Variable**	**Group**	**Before the intervention**	**3 months after the intervention**	***p*-value**
**Child Weight (gram)**	experimental	7338 ± 652.37	8330.84 ± 995.75	0.001
	control	7582 ± 639.189	8020.91 ± 916.049	0.001
	*p*-value	0.069	0.001	

## Discussion

The results of this study showed a significant increase in the mean score of knowledge, HBM constructs exclude Perceived barriers(decrease in the mean score of Perceived barriers), feeding behaviors, and weight of children in the experimental group three months after the educational intervention.

Also, knowledge of mothers in the experimental group increased significantly three months after the intervention, which is consistent with the results of studies by Anjomshoa et al. [27], Rolling et al. [28], Aghdasi et al. [20], and Mulualem et al. [29]. The more mothers are aware of the effects and causes of growth disorders and how to feed the child properly, the better their performance in preventing the child from developing growth disorders. This increased awareness is possible with education which plays an important role in prevention of children growth disorders.

Perceived sensitivity in the mothers of the experimental group significantly increased after the educational intervention, indicating the effectiveness of intervention in improving perceived sensitivity of the experimental group, which consistent with the results of studies by Kashfi et al. [14] and Navabi et al. [19]. The educational intervention for experimental group is performed in educational sessions by giving presentations, questions and answers, group discussions, informative posters, brochures, and video clips that increased the perceived sensitivity in people.It is while, in a study by Hazavei et al., the perceived sensitivity was not significantly different between the experimental and control groups [6]. which can be due to the difference in the study environment and how the intervention is implemented.

In the present study, the perceived severity in the experimental group increased significantly after the intervention, indicating the mother’s success in improving weight of the children, thanks to the knowledge on the grave effects of growth disorders, which is consistent with the result of studies by Kamal et al. [30] and Alizadeh Siuki et al. [31]. The perceived severity of growth disorders in children refers to the fact that mothers are well aware of the severe consequences of growth disorders in their children, both in the long- (thinness, short stature, low weight) and in short-term (severe anemia, recurrent infectious diseases, etc.) and they know that growth disorders, particularly chronic disorders, may have grave effects on the life of their child And In one of the sessions, The mother of an 8-month-old child who had a growth disorder was invited and she spoke to people about the growth disorder and the problems caused by it. In this meeting, it was emphasized to change the positive attitude of people to prevent growth disorders and the role of mothers in proper feeding of children that increased the perceived severity in people.

In the present study, the perceived benefits showed a significant increase in the experimental group after the intervention, which is consistent with the results of studies by Navabi et al. [19], Matlabi et al. [32] and Larijani. et al. [33] presenting educations about growth disorder and educational posters, brochures, and video clips help the increase of perceived benefits. The perceived benefits means that mothers are aware of the benefits of preventing their child from developing a growth disorder, benefits that lead to the normal growth of weight, height, and head circumference in children under one year of age and thus prevention of the treatment costs and improvement of children and their family quality of life.

In the present study, the perceived barriers showed a significant decrease in the experimental group after the intervention. This is consistent with the results of studies by Diddana et al. [34], Elfeshawy. et al. [35] and Vahedian-Shahroodi. et al. [36].

Perceived barriers are one’s belief in the costs of a new behavior. after the educational intervention, experimental group learn about the benefits of preventive behaviors from growth disorder and have less barriers for taking these behaviors. In current study, presenting educations about growth disorder and educational brochures help the increase of perceived benefits and dominating on barriers.

In the present study, the mean score of self-efficacy in the experimental group significantly increased after the intervention. This is consistent with the results of studies by Alizadeh Siuki et al. [31], Navabi et al. [19], Prasetyo et al. [37] and Gupta et al. [38]. that indicating the effect of the educational intervention on the self-efficacy of the mothers, i.e. the training of the mothers in the experimental group led them to believe that they could take good care of their children, use the health advice of the staff effectively, and prepare the proper food for their child and according to the findings of this study, the more satisfied mothers are with their self-adequacy, the better their child will grow.Studies show that people with high perceived self-efficacy are more committed to activities in times of challenge and difficulty and spend more time and effort doing activities [39].

In the present study, the mean score of cues to action(health center staff, physicians, the Internet, and the mass media) in the experimental group significantly increased after the intervention. The results of other studies are consistent with the results of this study [40, 41] that indicating the effectiveness of the intervention in choosing proper cue to action by mother, which is consistent with the results of study by Hazavei et al. [6].

In the present study, the feedind behavior showed a significant increase in the experimental group after the intervention.The results of Arikpo et al. [42], Hazavehi et al. [6] and Salavati Ghasemi et al. [43] also showed the effect of education on improving complementary feeding methods in children under 24 months. In the study of Rolling et al., Educational intervention based on social cognitive theory had an effective role on children's nutritional behavior [28]. that indicating the effect of education on mothers’ feedind behavior after the educational intervention. In the study of Golshiri et al., there was no significant difference between the mean score of mothers' performance regarding children's growth in the two groups of lecture and self-study in the three months after education [44] which could be due to the difference in the teaching methods of the two studies.

In the present study, weight of children in the experimental group significantly increased after the intervention, which is consistent with the results of studies by Kashfi et al. [14], Aghdasi et al. [20], Salavati Ghasemi et al. [43] and Mazani et al. [45].

In a study by Miller et al., training mothers had the greatest impact on child growth [46].

In a study by Cheng et al. in Nepal, showed that women and mothers' education had the greatest impact on children's growth. Training was found to be a factor influencing the growth of a 12-month-old child, and that early childhood education could improve the physical growth of children at later ages [47]. In this study, the educational intervention increased the constructs of the health belief model, except for the barriers perceived in mothers, and also the behavior of feeding children improved in mothers, which can be effective in increasing children's weight. Theory-based educational intervention It is one of the strengths of the present study. Also, the follow-up of the clinical effect of the current intervention by examining weight changes in the two intervention groups and the control group at two time intervals before and 3 month after the intervention is another strength of the present study.

### Limitations

This study had some limitations, including the fact that economic status of families is one of the factors affecting the growth of children that was not under the control of the researcher. This debate has been somewhat alleviated by discussions about suitable nutritional substitutes with the same nutritional value but cheaper cost. Difference of participants’ intelligence quotient, cognitive and psychological level was another limitation.

## Conclusion

Based on the results, the educational intervention based on the HBM improved the knowledge and feeding behavior of mothers and improved Growth Disorders of child. This study showed the educational intervention based on the HBM improved Hence, this model can act as a framework for designing and implementing educational interventions for prevention of growth disorders in 6–12 month children. Monitoring the regular growth of children from birth, increasing public knowledge through mass media, and involving husbands play an important role in preventing growth disorders. However, to accurately assess the success and survival rates, further studies on mothers of children with short stature (indicating chronic malnutrition) or mothers of children with growth disorder over one year of age (given that the effect of food compared to breast milk on child growth increases after one year of age) are highly recommended. 

## Data Availability

The datasets generated and/or analysed during the current study are not publicly available due to limitations within the ethics approval but are available from the corresponding author on reasonable request.

## Abbreviation

HBM: Health Belief Model
